# Liver resections for metastasis: surgical outcomes of a single center academic institution

**DOI:** 10.1186/s12893-020-00920-7

**Published:** 2020-10-27

**Authors:** Phillipe Abreu, Raphaella Ferreira, Danilo Saavedra Bussyguin, Eduardo DaCás, Vighnesh Vetrivel Venkatasamy, Flávio Daniel Saavedra Tomasich, Luiz Arnaldo Szutan

**Affiliations:** 1 Erasto Gaertner Hospital, Centro de Projetos de Estudo E Pesquisa (CEPEP), Curitiba, PR Brazil; 2 Faculdade de Ciências Médicas da Santa Casa de São Paulo, Departamento de Cirurgia, Área de Fígado e Hipertensão Portal, São Paulo, SP Brazil; 3 Department of Surgery, University of Miami, Miami Transplant Institute, Jackson Memorial Hospital, Miami, FL USA; 4 Hospital Erasto Gaertner, Curitiba, PR Brazil

**Keywords:** Liver resection, Hepatectomy, Liver tumors, Liver metastases, Postoperative complications

## Abstract

**Background:**

Hepatic metastasis are frequent and liver resection may be an option for some cases, despite the high complexity of the procedure and the possibility of postoperative complications.

**Methods:**

This retrospective comparative descriptive study aims to evaluate a series of 86 consecutive liver resections (LRs) performed for the treatment of metastatic liver tumors, comparing the results between patients undergoing major and minor LR. All patients submitted to LR from October 2010 to July 2015 at the Erasto Gaertner Hospital in Curitiba-PR were included. Quantitative numerical variables were analyzed with the Student t-test. The nonparametric Mann–Whitney U test was used for numerical variables of non-normal distribution. Categorical variables were analyzed with the Chi-square test with Fisher's correction. The data were analyzed with the SPSS 23.0 and STATA 15 programs, being p < 0.05 considered statistically significant.

**Results:**

Eighty-six LR were performed, 56 cases by colorectal metastasis. The major LR corresponded to 68 cases, with 13.2% of Clavien-Dindo III–V complications and 2.9% of reoperation rate. Eighteen minor LR were performed and one patient had a postoperative complication requiring reoperation.

**Conclusion:**

Preoperative elevation of transaminases and jaundice negatively influence surgical outcomes in patients undergoing LR. Tumors greater than 3 cm presented worse postoperative survival. Major LR did not significantly increase the surgical morbidity rate.

**Institutional Review Board registration:**

1.122.319/2015

## Background

Secondary hepatic tumors are frequent because the liver is the main solid organ affected by hematogenic metastases [1]. The main origins of these metastases are cancers of the colon, pancreas, ovary, rectum, stomach, lungs and kidneys [1]. Liver resection (LR) is an option to treat colorectal metastases with a 5-years overall survival of approximately 70% [2]. Neuroendocrine tumors liver metastases can also be treated with LR, with a 5-year survival of 60 to 80% [3].

There is a progressive trend in the indication of surgical approaches in hepatic metastases, due to the advance in tumor response to chemotherapy treatment, allowing the resection of tumors that initially exceeded the limits of resectability [4].

Liver surgery for the treatment of colorectal metastasis should aim at R0 resection, saving two segments adjacent to the resected segment and allowing the inflow and outflow of independent blood and bile drainage [5]. The future liver remnant should have no less than 25 and 30% of the total liver volume in cirrhotic and noncirrhotic patients, respectively [6].

Currently, there is a tendency to perform parenchymal-spearing LRs, obtaining oncologic resection with minimal margins, decreasing the risk of postoperative liver failure and allowing future LRs when necessary [7].

The extent of LR is based on the preoperative evaluation of the patient. Residual parenchyma, presence of portal hypertension and hypoalbuminemia should be evaluated [8]. Patients with non-cirrhotic livers can tolerate a resection of up to 75% of its volume or up to 6 segments, but patients with Child–Pugh B or C livers have high rates of complications even when undergoing minor LRs [9]. Therefore, the preoperative evaluation should be individualized according to the particularities of each case [8].

The surgical morbidity of LRs varies in different studies due to differences in the categorization of complications, being estimated between 4.1 to 47.7% [10]. Clavien-Dindo's classification was described in 2004 with the aim of standardizing the evaluation of postoperative complications in an objective and reproducible manner [11]. It consists of five degrees: I-any deviation from normality postoperatively, without the need for surgical, endoscopic, radiological or pharmacological interventions (except anti-emetic, antipyretic, analgesic, diuretic and electrolyte drugs); II-need for pharmacological treatment (beyond that allowed in degree I); III-need for surgical, endoscopic or radiological intervention; IV-life-threatening complications; V-death [11].

The most dramatic of the complications described is the postoperative acute liver failure, related to the amount of liver tissue removed and previous liver function [12]. Biliary fistula has a prevalence of 4 to 17%. Post-operative bleeding may be present in up to 8% of hepatectomies [5]. Several thromboembolic phenomena are cited, such as venous thrombosis and pulmonary thromboembolism, as well as thrombosis of hepatic vein and portal vein [13]. Other complications can include acute renal failure, ascites, wound infection, intrahepatic abscesses and pneumonia [14]. LR has high technical complexity and should be performed in specialized and reference centers [12].

## Methods

This retrospective comparative descriptive study aims to evaluate the surgical outcomes of hepatectomies performed in an oncologic teaching hospital affiliated to the Brazilian Unified Health System. All patients submitted to LR from October 2010 to July 2015 at the Erasto Gaertner Hospital in Curitiba-PR, Brazil were included. Written informed consent form was obtained from all subjects. The project was approved by the institutional review board under the number 1.122.319/2015. All methods were carried out in accordance with relevant guidelines and regulations.

### Surgical indications

Resection in metastatic disease of tumors of several primary etiologies, which met criteria of anatomic resectability, after good response to systemic treatment, was indicated. Liver metastasis diagnosed at the same time or up to 6 months after the diagnose of the primary tumor were considered as synchronic metastases. Liver metastasis diagnosed after 6 months of primary diagnosis were considered as metacronic metastases. As a routine, we do not perform liver resections in the same surgical procedure as the primary tumor resection, due to increased morbidity and mortality when compared to staged resections [15, 16], although safely performed in selected centers [17–19]. The order of resection to be performed is determined by the patient’s symptoms, treating first the most symptomatic tumor.

### Diagnostic and preoperative workup

The preoperative evaluation of the cardiovascular, pulmonary, renal, hepatic, nutritional and anesthetic systems was performed in all patients undergoing LR, and may have some variation depending on the underlying disease. Recent imaging (less than 45 days) is mandatory to perform the procedure.

### Study variables

The clinical characteristics of the patients and the underlying disease, Performance-Status (PS), laboratory tests of liver function, type of liver resection, associated surgical procedures, need for transfusion, length of stay, mortality and postoperative complications following the Clavien-Dindo classification were collected and analyzed.

### Characteristics of the surgical procedure

Vast majority of the cases were open Liver resections, only 4 cases performed by minimally invasive technique. All open resections were performed with intra-operative sonographic analysis of the lesions and careful delimitation or their limits with ultrasound assessment. In all cases hilar vascular control was performed by intermittent Pringle Maneuver (15–20 min clamped dissection, followed by 15–20 min of unclamped dissection). Patients were transferred to Intensive Care Unit (ICU) of the hospital for postoperative care.

### Study groups

The patients were divided into groups according to the surgery performed. Group I = major LR (resection of at least 3 liver segments or resection involving the segment IV) and Group II = minor LR. Minor resections included anatomic resections and wedge resections. Major resections included central, right and left resections, depending on the tumor locations. Due to the nature of intra-hepatic metastatic spread, there was a high heterogeneity of the procedure performed, thus the option of recording the data according to the number of segments resected reflected as the best option to standardize groups for analysis in relation to the hepatic volume.

### Statistical analysis

The data were expressed as mean and standard deviation or as median and interquartile range for non-normal distribution. Quantitative numerical variables were analyzed with the Student t-test. The nonparametric Mann–Whitney U test was used for numerical variables of non-normal distribution. Categorical variables were analyzed with the Chi-square test with Fisher's correction. The data were analyzed with the SPSS 23.0 and STATA 15 programs, being p < 0.05 considered statistically significant.

*Survival analysis* A survival sub-analysis was performed with a univariable comparison. The factors that were taken into consideration in this analysis are: the presence of preoperative jaundice, the presence of high values of transaminases (AST and ALT) in the preoperative period, the type of resection performed (greater or lesser) and the size of the injury (greater or lesser than 3.0 cm).

## Results

In the period analysed, 86 LRs were performed. The median age of the patients was 56.1 years (IQR 45.9–65.8). Thirty-eight (44.2%) patients were male. Other epidemiological data are presented in Table 1.

**Table 1:** Demographic characteristics and epidemiological profile of the sample

Variable	Total (n = 86)	Colorectal (n = 56)	Non-colorectal/non-neuroendocrine (n = 30)	p
Death rate, number (%)	42 (48.8)	29 (51.8)	13 (43.3)	0.45
Recurrence rate, number (%)	48 (55.8)	34 (60.7)	14 (46.7)	0.21
Median follow-up, years (IQR)	2.4 (0.9–5.1)	2.6 (1.0–5.0)	2.0 (0.2–6.7)	0.71
Sex, male (%)	38 (44.2)	24 (42.9)	14 (46.7)	0.73
Age, median years (IQR)	56.1 (45.9–65.8)	58.4 (46.6–65.8)	52.0 (43.2–63.1)	0.24
ECOG-PS, number (%)				0.002
0	17 (19.8)	11 (19.6)	6 (20.0)	
1	58 (67.4)	43 (76.8)	15 (50.0)	
2	11 (12.8)	2 (3.6)	9 (30.0)	
BMI (kg/m^2^), (IQR)	25.0 (23.5–27.6)	25.2 (23.5–27.7)	25.0 (23.0–26.7)	0.84
History of smoking, number (%)	33 (41.8)	21 (42.9)	12 (40.0)	0.80
Smoking, packs-year (IQR)	24.0 (13.7–38.5)	20.0 (7.0–39.5)	30.0 (21.5–36.5)	0.27
Hypertension and/or heart disease, number (%)	32 (37.2)	23 (41.1)	9 (30.0)	0.31
Family history of cancer, number (%)	13 (20.3)	9 (21.4)	4 (18.2)	0.75
Differentiation grade, number (%)				0.02
Well differentiated	25 (29.1)	11 (19.6)	14 (46.7)	
Moderately different	48 (55.8)	36 (64.3)	12 (40.0)	
Poorly differentiated	13 (15.1)	9 (16.1)	4 (13.3)	
Free margins, number (%)	77 (89.5)	47 (83.9)	30 (100.0)	0.02

*IQR* interquartile range, *ECOG-PS* Eastern Cooperative Oncology Group - Performance Status, *BMI* Body Mass Index

Sixty-eight (79.1%) major hepatectomies (group I) and 18 (20.9%) minor hepatectomies (group II) were performed. Group I presented 48 patients (72.7%) with ASA 2, while group II presented 13 (76.5%) (p = 0.93). The mean time of surgery was 205 min (IQR 122.5–300.0) in group I and 180 min (IQR 120.0–300.0) in group II (p = 0.49).

The overall rate of postoperative complications Clavien-Dindo III or higher was 11.6% with 9 patients (13.2%) in Group I and 1 patient (5.6%) in Group II (p-value = 0.36). The global rate of reoperation was 3.5%, 2 patients (2.9%) of group I and 1 patient (5.6%) of group II (p = 0.59).

Group I presented 56 surgeries (82.4%) with anatomical resection, while Group II presented 5 (27.8%), with significant statistical difference (p < 0.001). The median admission in group I was 5 days (IQR 3.0–7.0) while in group II was 3 days (IQR 3.0–6.0) (p = 0.15). The mean time of ICU was 2 days (IQR 1.0–3.0) in group I and 2 days (IQR 0–2.2) in group II (p = 0.12). There was no difference in the use of hemoderivatives between the groups, being 3 units of RBC concentrate in group I (IQR 2.0–4.0) and 3.5 in group II (IQR 2.0–6.0) (p = 0.56).

Among the 86 LRs performed, 56 (65.1%) were indicated by metastases of colorectal tumor, and 47 (69.1%) were treated with major hepatectomy. Surgical outcomes are presented in Table 2.

**Table 2:** Surgical data—major LR versus minor LR

Variable	Total (n = 86)	Major LR (n = 68)	Minor LR (n = 18)	p
ASA, number (%)				0.93
1	10 (12.0)	8 (12.1)	2 (11.8)	
2	61 (73.5)	48 (72.7)	13 (76.5)	
3	12 (14.5)	10 (15.2)	2 (11.8)	
Hemotransfusion, number (%)	30 (34.9)	24 (35.3)	6 (33.3)	0.87
PRBC, units (IQR)	3.0 (2.0–4.0)	3.0 (2.0–4.0)	3.5 (2.0–6.0)	0.56
Surgical time, minutes, median (IQR)	200.0 (120.0–300.0)	205.0 (122.5–300.0)	180.0 (120.0–300.0)	0.49
Pos-operative complications Clavien III–V, number (%)	10 (11.6)	9 (13.2)	1 (5.6)	0.36
Reoperation, number (%)	3 (3.5)	2 (2.9)	1 (5.6)	0.59
Lenght of stay, days, median (IQR)	4.0 (3.0–6.2)	5.0 (3.0–7.0)	3.0 (3.0–6.0)	0.15
ICU time, days, median (IQR)	2.0 (1.0–3.0)	2.0 (1.0–3.0)	2.0 (0.0–2.2)	0.12
Lesion size, cm (IIQ)	3.5 (2.7–5.0)	3.5 (2.7–5.0)	4.0 (3.0–5.0)	0.54
Number of lesions (IQR)	1.0 (1.0–3.0)	1.0 (1.0–2.0)	1.0 (1.0–1.2)	0.57
Vascular involvement, number (%)	23 (26.7)	18 (26.5)	5 (27.8)	0.91
Colorectal tumor, number (%)	56 (65.1)	47 (69.1)	9 (50.0)	0.13
First LR, number (%)	77 (89.5)	61 (89.7)	16 (88.9)	0.92
Anatomical ressection, number (%)	61 (70.9)	56 (82.4)	5 (27.8)	< 0.001
Synchronic tumor, number of patients (%)	18 (32.1)	16 (23.5)	2 (11.1)	0.48

*IQR* interquartile range, *ASA* American Society of Anesthesiologists, *ICU* Intensive Care Unit, *LR* liver resection, *PRBC* packed red blood cell

**Fig. 1:**
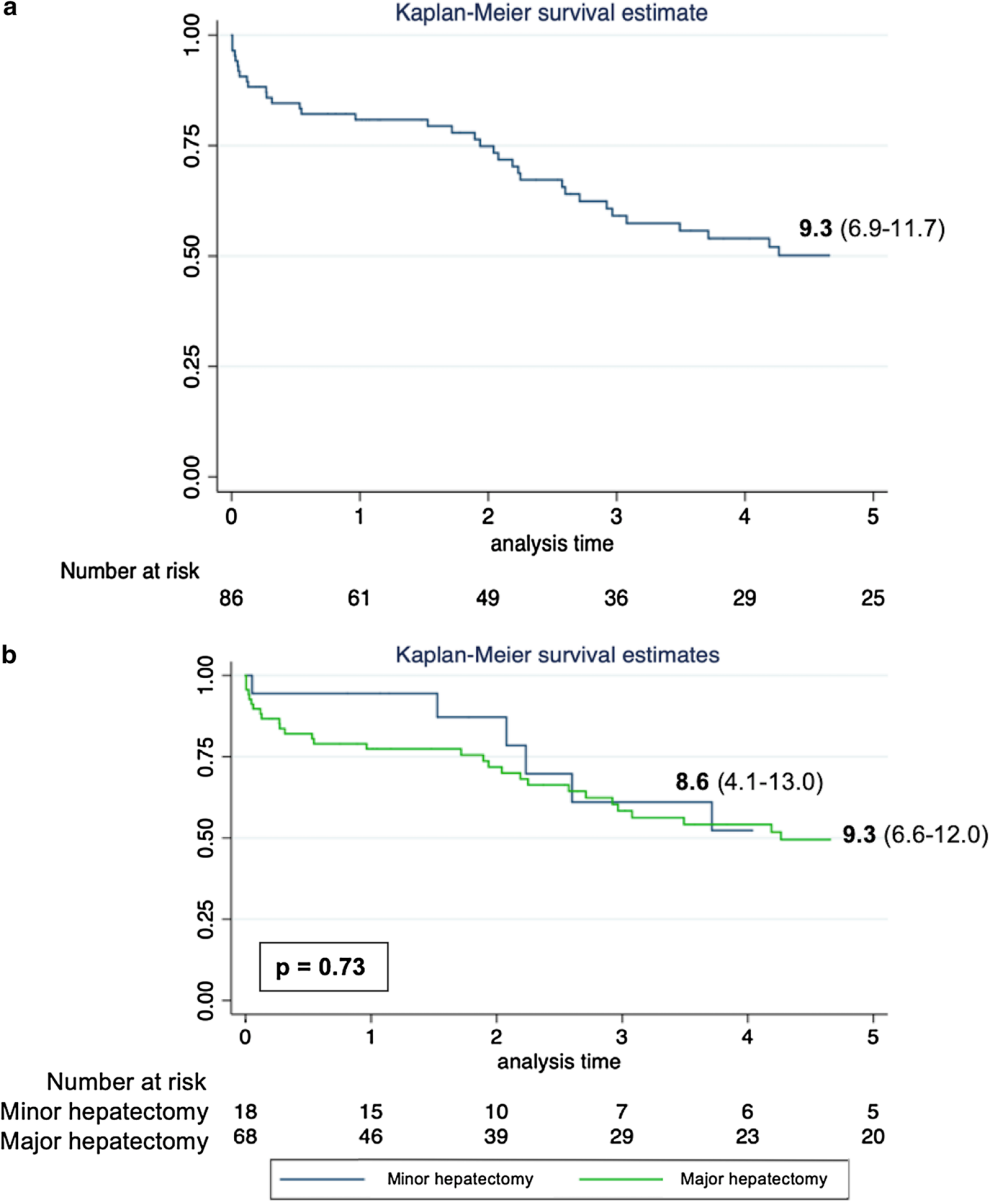
Overall Survival in years. **a** Complete cohort. **b** Stratified by the type of LR performed

The mean actuarial global survival rate over 1, 3 and 5 years was 69.7%, 40.6% and 27.9%, respectively (Fig. 1a). Group I presented a mean survival rate of 9.3 years (IQR 6.6–12.0) and Group II 8.6 years (IQR 4.1–13.0) without significant difference (p = 0.73) (Fig. 1b).

**Fig. 2:**
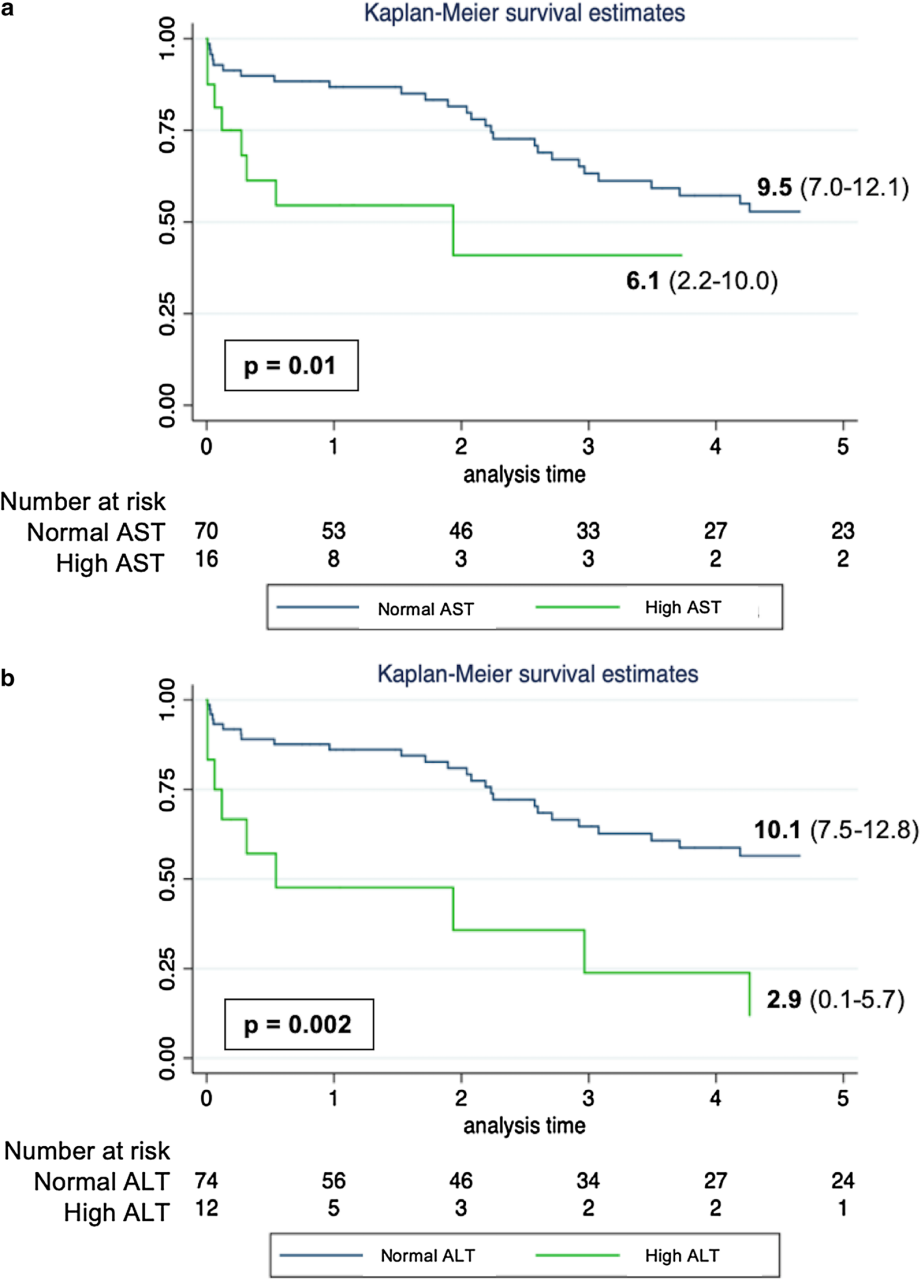
Overall Survival in years by groups. **a** Stratified by preoperative values of AST. **b** Stratified by preoperative values of ALT

The preoperative elevation of transaminases (AST and ALT) showed significant influence on the mean survival of the sample. Patients with high preoperative AST had a mean survival of 6.1 years (IQR 2.2–10.0), while those with normal AST values achieved a mean survival of 9.5 years (IQR 7.0–12.1), (p = 0.01) (Fig. 2a). In respect to the ALT values, the group with preoperative elevation achieved a mean survival of 2.9 years (IQR 0.1–5.7) compared to 10.1 years (IQR 7.5–12.8) in the group with preoperative ALT normal values, (p = 0.002) (Fig. 2b).

**Fig. 3:**
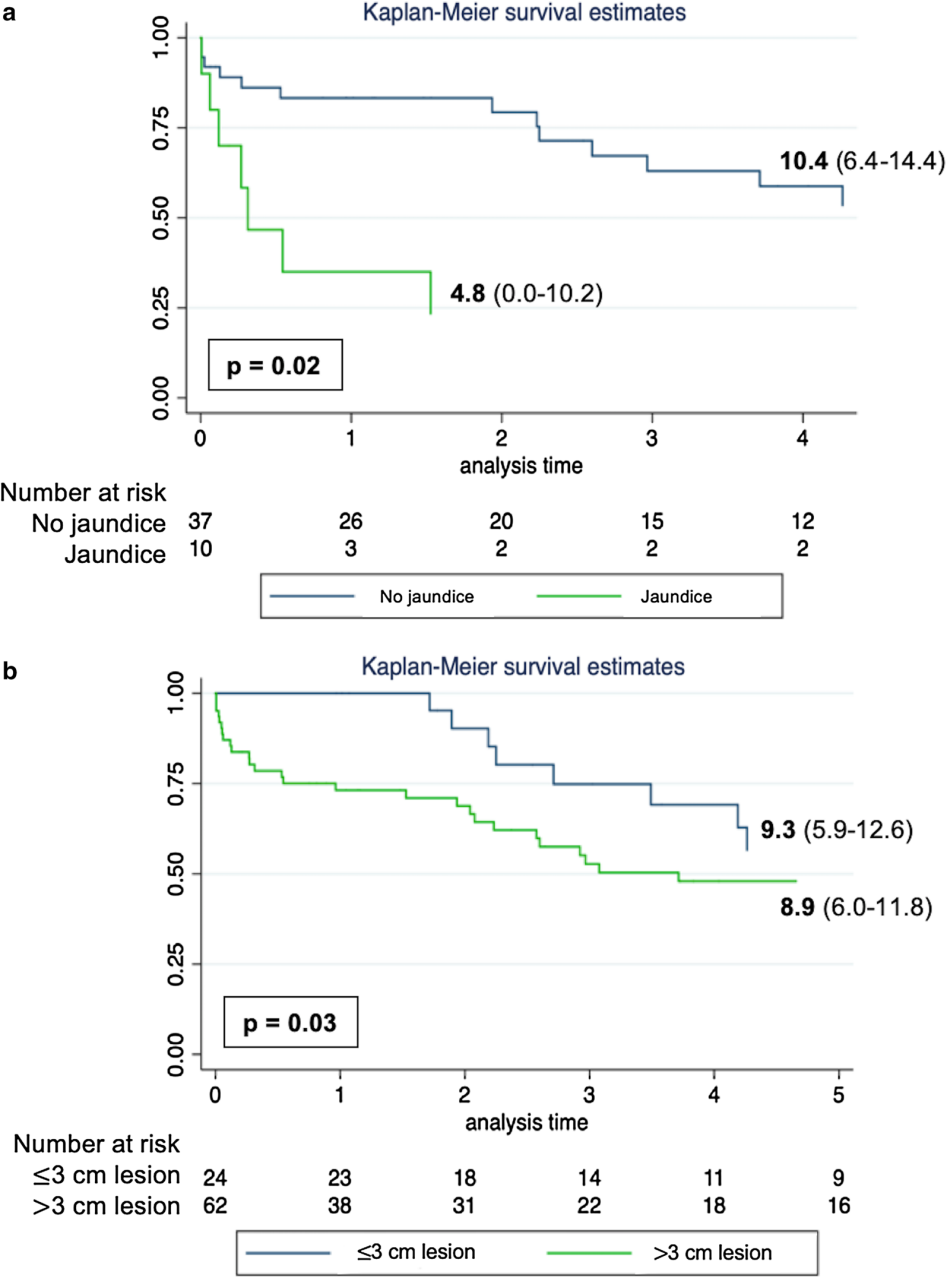
Overall Survival in years by groups of interest. **a** Stratified by the presence of preoperative jaundice. **b** Stratified by tumor size in centimeters

When survival is compared between the groups with and without preoperative jaundice a significant difference is obtained (p = 0.02), with the non-jaundice group having a mean survival of 10.4 years (IQR 6.4–14.4) and the group with preoperative jaundice 4.8 years (IQR 0.0–10.2) (Fig. 3a).

The size of the lesion showed significant influence on survival (p = 0.03). Patients with tumors of up to 3.0 cm had a mean survival of 9.3 years (IQR 5.9–12.6), while those with tumors greater than 3.0 cm had a mean survival of 8.9 years (IQR 6.0–11.8) (Fig. 3b).

## Discussion

Improved surgical technique and better knowledge of the physiological anatomy of the liver, combined with better diagnostic conditions, allowed LR to be performed more often in oncologic patients [20].

The largest indication for surgery in our sample was liver metastasis of colorectal cancer (65.1% of cases). This number is in line with the current progressive trend to operate patients with colorectal metastasis due to the advance in chemotherapy treatment, which can provide a 50% survival over 5 years [2, 4]. Major LR was the surgical approach used in these patients to perform a R0 resection.

The surgical outcomes of LRs are also described in other studies, such as Resende et al. which showed a rate of postoperative complications of 11.4%, including non-oncologic cases [21]. Other authors such as Amico et al. show complication rates of 14.7%, highlighting intraperitoneal collection, pleural effusion and hemorrhage [20]. In both national studies there was a higher rate of postoperative complications in patients undergoing major LRs.

Our study presented an overall rate of postoperative complications of 11.6%, even including a large percentage of major LRs (79.6%)—higher than the percentages found in other Brazilian series, with 31.4% and 43.2% [20, 21]. Considering that the complication rates have progressive increase according to the extent of LR, these values are in line with those found in literature [8].

Complication rates are also related to functional disability of the liver, and patients with Child–Pugh B or C scores have high incidence of postoperative complications, limiting in some cases the performance of major LRs [9]. Since our sample included, in its majority, patients operated for colorectal metastasis and not for hepatocellular carcinoma (a condition that predisposes to liver cirrhosis and functional limitation of the liver), it is justified to perform major LRs in 69.1% of these cases.

However, patients with hepatic functional changes (preoperative elevation of AST, ALT and bilirubin) presented significantly more postoperative complications in our sample, which reinforces the need for careful prior evaluation of future liver remnant [22]. Tian et al. analyzed 74 patients undergoing LR for hepatocellular carcinoma and concluded that the presence of preoperative jaundice is directly related to the patient's prognosis [23].

Lesions of larger diameters in general require more extensive resections, which can lead to further postoperative complications. The difference in the number of complications between major and minor LRs is described by Virani et al., but in our analysis there was no significant difference between the two groups (p = 0.36) [24]. It is worth noting that only one patient undergoing minor LR presented postoperative complications with the need for surgical reopening. The sample size did not allow a statistically significant difference in the rate of complications between major and minor LR, but with the expansion of the study it is likely that this difference is confirmed, following the pattern in the literature [24]. However, we found a significant difference in survival of patients with tumors larger than 3 cm, which showed no direct correlation with the type of resection performed.

While the overall rate of reoperation described in the literature is 5.2% [24], our sample presented an overall rate of only 3.5%, demonstrating technical suitability of the service.

We understand as limitations of our study as uncontrolled retrospective characteristic. There are also limitations of technological resources due to the work being performed in a public Unified Health System teaching hospital where there are budgetary restrictions.

## Conclusion

We can conclude that preoperative elevation of transaminases and jaundice negatively influence surgical outcomes in patients undergoing LRs. Tumors greater than 3 cm presented worse postoperative survival. Major LR did not significantly increase the surgical morbidity rate.

## Data Availability

The datasets used and analysed during the current study are available from the corresponding author on reasonable request.

## Abbreviations

ALT: Alanine aminotransferase
ASA: American Society of Anesthesiologists
AST: Aspartate aminotransferase
BMI: Body Mass Index
ECOG-PS: Eastern Cooperative Oncology Group - Performance Status
ICU: Intensive Care Unit
IQR: Interquartile range
LR: Liver resection
PRBC: Packed red blood cell
